# Multiple Micronutrient Supplementation during Pregnancy and Increased Birth Weight and Skinfold Thicknesses in the Offspring: The Cambridge Baby Growth Study

**DOI:** 10.3390/nu12113466

**Published:** 2020-11-12

**Authors:** Clive J. Petry, Ken K. Ong, Ieuan A. Hughes, David B. Dunger

**Affiliations:** 1 Department of Paediatrics, University of Cambridge, Cambridge CB2 0QQ, UK; Ken.Ong@mrc-epid.cam.ac.uk (K.K.O.); iah1000@cam.ac.uk (I.A.H.); dbd25@cam.ac.uk (D.B.D.); 2 MRC Department of Epidemiology, University of Cambridge, Cambridge CB2 0SL, UK; 3 Institute of Metabolic Science, University of Cambridge, Cambridge CB2 0QQ, UK

**Keywords:** adiposity, development, fetal growth, gestational diabetes, minerals, vitamins

## Abstract

Multiple micronutrient supplementation (MMS) in pregnancy has previously been associated with positive effects on fetal growth, but its value in high-income countries remains controversial. In this study, we investigated effects of pregnancy MMS on offspring size at birth and adiposity, along with risks of various maternal outcomes of pregnancy, using the prospective Cambridge Baby Growth Study. Maternal MMS was reported in 528 out of 970 women who completed pregnancy questionnaires. Gestational diabetes (GDM) was assessed using results from 75 g oral glucose tolerance tests at week 28 of pregnancy. Offspring size at birth was assessed using standard anthropometric measurements and adiposity using skinfold calipers. MMS was associated with increased risk of developing GDM (risk ratio = 1.86 (1.13–3.08), *p* = 0.02), as well as increased offspring size at birth in terms of weight (*p* = 0.03), head circumference (*p* = 0.04), and flank, and subscapular and triceps skinfold thicknesses (*p* = 0.04, 0.03, and 0.003, respectively). There was no association with quadriceps skinfold thickness (*p* = 0.2), suggesting that the increased adiposity was partially regionalized. In women who underwent oral glucose tolerance testing, nearly all of these associations were attenuated by adjusting for GDM. These results suggest that the increased offspring size at birth, including (regionalized) adiposity associated with pregnancy, and MMS may be partially related to the development of GDM.

## 1. Introduction

Multiple micronutrient supplementation in pregnancy, as shown in a recent Cochrane review covering clinical trials that included a total of over 141,000 participants [1], leads to a number of positive effects in pregnancy, including a lowering of the prevalence of low birth weight babies, numbers born small for gestational age (SGA), and the prevalence of preterm deliveries. Nineteen of the twenty clinical trials included in this analysis took place in low- or middle-income countries where multiple micronutrient deficiencies are prevalent during pregnancy. The remaining trial was conducted in the UK, where a low-income population with nutritional deficiencies was studied, despite being in a high-income country [2]. This trial found no effect of supplementation on low birth weight, SGA, or preterm birth rates. However, it did report a reduced prevalence of anemia in pregnancy [2]. In contrast to the numbers of studies in low- and middle-income countries [3], formal studies are rarely undertaken in high-income countries [4], such as the UK, where pregnant women may be better nourished [5]. Women from these countries may be more likely to be nutrient replete, running the risk of adverse consequences arising from supplementation due to nutrient overload [6]. However, individual women from such countries can still be undernourished or micronutrient deficient [7].

In this analysis, we investigated the potential effects of multiple micronutrient supplementation on adverse maternal outcomes and offspring growth in a relatively contemporary pregnancy and birth cohort recruited from a single center in Cambridge, UK [8]. This cohort came from a high-income country, and indeed, the participants were generally less deprived than the national average for this country [8]. The maternal participants also appeared to consume varied diets [9]. In the absence of having detailed records of the nutritional status for this cohort, it should be noted that, in the UK as a whole, at the start of recruitment in this cohort, younger adults (particularly women with low socioeconomic statuses) showed relative trends for low micronutrient intakes [10].

## 2. Materials and Methods

### 2.1. Cambridge Baby Growth Study

The longitudinal Cambridge Baby Growth Study (CBGS) recruited women (and their partners and offspring) attending early pregnancy ultrasound clinics at the Rosie Maternity Hospital, Cambridge, UK between 2001–2009 [8]. This observational study was run prospectively (e.g., the collection of data relating to size at birth and gestational diabetes (GDM)). However, in order to gain as much useful information as possible, the measurements were supplemented with data collected retrospectively (by one of the co-authors) from the participants’ hospital notes (e.g., those relating to blood pressure outcomes). In addition, the index of multiple deprivation was derived from the postcode of the participants’ home addresses as described [11]. In this cohort, 95.3% of the offspring were white, 1.7% were mixed race, 1.3% were black (African or Caribbean), and 1.7% were Asian.

Although 2229 pregnant women were originally recruited to the cohort, all of whom were over 16 years of age and able to give consent, 571 of them withdrew prior to the birth of their baby and self-excluded from the study. From all of the women recruited to the CBGS during the years 2001–2009, the following were excluded from the present analysis: those who gave birth to twins (because of the impact of multifetal pregnancies on offspring size at birth), those that had already withdrawn from the study before the birth of their baby, and those that did not fill in and return their pregnancy questionnaire (specifically the question about supplement intake during pregnancy).

### 2.2. Ethical Approval

The CBGS was granted ethical approval by the Cambridge Local Research Ethics Committee, Addenbrooke’s Hospital, Cambridge, UK (00/325). All procedures followed were in accordance with the institutional and international guidelines. Written informed consent was obtained from all women (on their own behalf and on behalf of their baby).

### 2.3. Assessment of Multiple Micronutrient Supplementation in Pregnancy

Each of the pregnant women were given an extensive questionnaire at recruitment to fill in as pregnancy progressed (with assistance from trained research nurses if required) [9]. These questionnaires were collected by the research nurses after the birth of the babies. The questions that were asked were wide-ranging, but as part of a section about lifestyle there was a question that read “Have you taken any dietary supplements during the pregnancy?” If this was answered in the affirmative, there was a table to fill in with “Name of Product”, “Frequency”, and “Gestational Weeks.” Multiple micronutrient tablets (defined as tablets containing at least three micronutrients) were entered as brand names; internet searches were subsequently performed (August–December 2019) by one of the investigators to confirm brand constituent vitamins and minerals. The questionnaires were completed by 1239 of the CBGS recruits, although not all participants provided unequivocal responses to the question about dietary supplementation (these women were excluded from the present analysis). Where a brand making multiple micronutrient supplements was listed without the full micronutrient tablet name being entered onto the questionnaire, an assumption was made that the supplement taken was the best-selling one from that brand. Exposed women were those who self-reported taking multiple micronutrients at any stage of pregnancy. Non-exposed women were those who did not supplement their diets with multiple micronutrients; they may either have not taken any dietary supplements, or they may have taken single micronutrients, such as folic acid and/or iron.

### 2.4. Food Frequency Intakes in Pregnancy

Food frequency intakes were also collected from the pregnancy questionnaires [9]. As part of a section of the questionnaire about lifestyle, there was a short (specific) food frequency questionnaire which covered most of the major food and drink types. Regarding food, the participants were asked, “How often did you eat the following foods during pregnancy?” and the response involved ticking one of the following options: “never”, “1–3 times per month”, “1–3 times per week”, “4–6 times per week”, or “once or more per day.” Regarding drinks, the participants were asked the number of times they drank a particular drink per day or per week (depending upon the likely consumption frequency of that drink).

### 2.5. Assessment of Pregnancy Outcomes

GDM was classified following overnight fasting and a standard 75 g oral glucose tolerance test (OGTT) around week 28 of pregnancy in 1074 of the women [12] using the International Association of Diabetes in Pregnancy Study Group diagnostic criteria [12,13]. Pre-eclampsia was classified using the terms “preeclampsia”, “pre-eclampsia”, “PET”, or “pre-eclamptic toxemia”, and was recorded from the hospital notes. It was clinically diagnosed using a combination of new-onset hypertension (systolic blood pressure > 140 mmHg and/or diastolic blood pressure > 90 mmHg) after week 20 of pregnancy and proteinuria (> 300 mg/day). Gestational hypertension was classified using a combination of hospital notes and blood pressure measurements in the second half of pregnancy, as described previously [14]. This was achieved in 720 of the women. In other women, the hospital notes were either not available to us or the hospital notes did not include blood pressure measurements from these pregnancies. Low birth weight was defined as an unadjusted birth weight of less than 2.5 kg. SGA was defined as a birth weight that was below the tenth percentile for gestational age against UK growth charts [15]. Premature birth was defined as one occurring before 37 weeks of gestation.

### 2.6. Assessment of Offspring Size at Birth

Birth weight was recorded from the hospital notes. Other newborn measurements (length, head circumference, and skinfold thickness at four sites) were made by trained pediatric nurses as soon as possible after birth (at a median (inter-quartile range) age of two (1–16) days). Each measurement was made three times and the mean value was used. Body length was measured (to the nearest 0.1 cm) using a SECA 416 Infantometer. Head circumference was measured using a tape measure. Triceps (posterior midline of upper left arm, halfway between the acromial process and the olecranon), quadriceps (also known as thigh, found using a vertical line over the quadriceps muscle at midline of the left thigh, halfway between the top of the patella and the inguinal crease), flank (also known as supra-iliac, the diagonal plane in line with the natural angle of the iliac crest taken in the posterior axillary line immediately posterior to the iliac crest), and subscapular (the oblique angle below the left scapula) skinfold thicknesses were measured in triplicate on the left side of the body using Holtain calipers (Chasmors Ltd., London, UK) [16]. Intra-observer technical errors of measurement were 0.4–2.8% for the skinfold thicknesses. Equivalent inter-observer values were 2.0–3.2%.

Body mass indexes (BMIs) were calculated as the height (or length for the newborn babies) divided by the (pre-pregnancy for the mothers) weight squared. Ponderal indexes at birth were calculated as the body length divided by the weight cubed.

### 2.7. Data Availability

The dataset generated and/or analyzed during the current study is available in the Apollo repository (https://doi.org/10.17863/CAM.54014).

### 2.8. Statistical Analysis

Risk ratios/relative risks (RRs) of adverse pregnancy outcomes by multiple micronutrient supplementation were analyzed using logistic regression. Potential confounding of significant associations by dietary intakes were investigated by adjusting these models for food frequency intakes. Continuous variables were analyzed in statistical models using linear regression, adjusted for confounders where appropriate. Where the dependent variable residuals were skewed, the models were analyzed with prior transformation of the data so that the residuals were normally distributed. Other categorical variables were analyzed using a χ^2^-test, Fisher’s exact test (as appropriate), or logistic regression. RRs were calculated using the Stata binreg function. Missing data were dealt with using case or listwise deletions, and *p* < 0.05 was considered statistically significant throughout. The statistical analyses were performed using either Stata (version 13.1; Stata Corp., from Timberlake Consultants Ltd., Richmond, Surrey, UK) or R (version 3.6.1; The R Foundation for Statistical Computing, Vienna, Austria).

### 2.9. Sensitivity Analyses

Women who were diagnosed with anemia supplemented their diets with (presumably prescribed) iron or folic acid. The various statistical analyses were also performed with women with anemia excluded to test whether the evident trends were also present in those women whose multiple micronutrient supplementation was unprescribed. Further sensitivity analyses were performed by excluding women over 30 years of age to assess whether evident trends had an age-related basis, and by excluding those women whose multiple micronutrient supplementation only began after they had undergone OGTTs, assess whether it was associated with the development of GDM rather than partially relating to a response to a diagnosis of GDM.

## 3. Results

### 3.1. Clinical Characteristics and Multiple Micronutrient Supplement Constituents

This analysis included 970 study participants (442 of whom did not supplement their diets with multiple micronutrients and 528 that did). Of those that did supplement, 293 participants took Vitabiotics Pregnacare, 206 took Sanatogen Pronatal, and 67 took Tesco Multiplus Pregnancy (some women supplemented with more than one type; the constituents of these multiple micronutrient supplements are shown in Table S1). A wide variety of other brands of supplements were taken by a smaller number of participants (data not shown).

Those women included in the analysis were largely representative of the CBGS study participants as a whole (Table S2). With the exception of the number of twin pregnancies, which were specifically excluded from the analysis, only smoking during pregnancy differed in prevalence (in this case being less common) between those included in the analysis than in those excluded from it. Smoking during pregnancy, however, which was uncommon in this cohort of mothers [8], was not associated with any key phenotype (data not shown).

Women who supplemented their diets with multiple micronutrients had very similar clinical characteristics to those who did not supplement their diets with multiple micronutrients (Table 1). The only trait that differed was the height, where the mean height was 1 cm taller in women who supplemented their diets with multiple micronutrients.

### 3.2. Associations with Maternal Micronutrient Supplementation in Pregnancy

**Figure 1:**
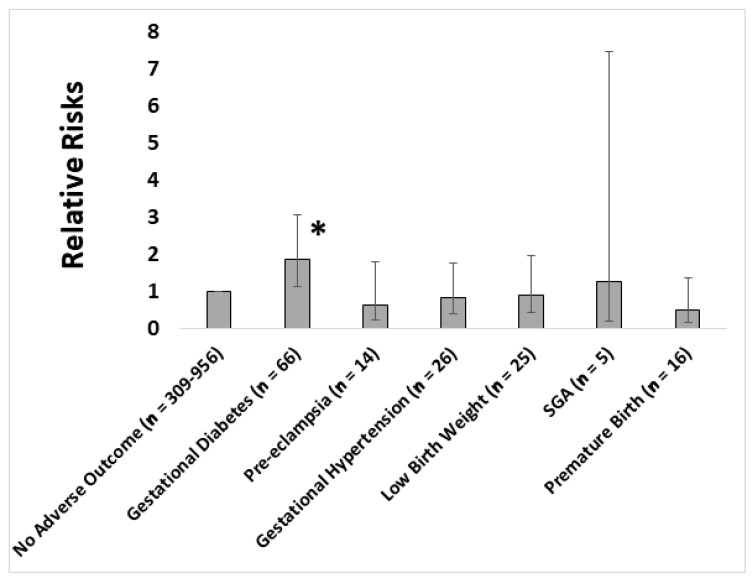
Relative risks of various adverse conditions of pregnancy in women who supplemented their diets with multiple micronutrients in pregnancy (* *p* < 0.05).

Of those women who supplemented their diets with multiple micronutrients, the majority of them started supplementing either before or at the start of pregnancy, and continued supplementing throughout pregnancy (Figure S1). Multiple micronutrient supplementation was strongly associated with parity, with proportionally fewer women supplementing as the parity increased (no/yes, in increasing order of parity: 163/258, 181/193, 67/55, 21/7, 1/2, and 1/1; *p* = 7.6 × 10^−5^). Supplementing with multiple micronutrients was also associated with an increased risk of developing GDM (*p* = 0.015; Figure 1). Excluding those participants whose multiple micronutrient supplementation did not start until after the OGTTs took place did not substantially alter the statistical relationship (RR = 1.87 (1.07–3.28); *p* = 0.03; n = 643). None of the food frequency intakes confounded this relationship to any great extent (Table S3). With the exception of GDM, multiple micronutrient supplementation was not associated with adverse pregnancy conditions or outcomes, such as pre-eclampsia (*p* = 0.4), gestational hypertension (*p* = 0.7), low birth weight (*p* = 0.8), SGA (*p* = 0.8), or premature birth (*p* = 0.2) (Figure 1).

Maternal multiple micronutrient supplementation in pregnancy was associated with a number of factors relating to increased size at birth and adiposity in the offspring (Table 2). This included increased birth weight, head circumference, and various skinfold thicknesses. Even the associations with measurements where statistical significance was not reached (e.g., with offspring BMI, ponderal index, and body length) were all in the same direction, that of an increased size at birth.

### 3.3. Subgroup Analysis in Women Who Underwent OGTTs

In women for whom OGTT data were available, except for the association with head circumference, all of the associations appeared attenuated by adjusting for GDM (Table 3). Even prior to this adjustment, all of the associations were already attenuated relative to those using the full dataset in this smaller subset of study participants.

### 3.4. Sensitivity Analyses

Significant associations with maternal GDM and increased offspring size at birth and adiposity were still evident even when women with anemia were excluded from the analyses (Table 4).

The increased risk of developing GDM associated with multiple micronutrient supplementation in pregnancy was still evident in mothers less than 30 years of age (Table 5). The number of women with adverse pregnancy outcomes who were less than 30 years of age were very low, as only a minority of pregnancies in the CBGS involved women in this age category. The only measure of adiposity that still showed a statistically significant association with multiple micronutrient supplementation in this small number of pregnancies was the subscapular skinfold thickness. Apart from associations with the offspring BMI and ponderal indexes, the standardized effect sizes of the other associations increased (in the same direction) relative to those in the full analysis, albeit without statistical significance in the small number of women tested.

## 4. Discussion

In this analysis, maternal multiple micronutrient supplementation in pregnancy in a population from a high-income country was associated with increased risk of GDM in the mother. It was also associated with results that are suggestive of increased offspring size at birth and adiposity. The consensus from previous studies in this area is that such supplementation is associated with reduced risk of offspring low birth weight and possibly reduced risk of the offspring being born SGA [1]. Consistent with a randomized trial that also took place in the UK [2], we could not replicate these associations in our population, which only had a relatively small number of low birth weight and SGA pregnancies. However, our associations are at least consistent with the idea that a generalized increased size at birth across a whole population could lead to a lower proportion of low birth weight and SGA pregnancies in offspring that otherwise would be close to the boundaries of definitions for these adverse outcomes of pregnancy.

Deficiencies in specific micronutrients, such as selenium, chromium, and zinc, have previously been shown to be associated with an increased risk of developing GDM [17]. Multiple micronutrient supplementation in pregnancy might therefore be expected to reduce the incidence of GDM, although it appears not to have been investigated before. In our population, however, it was associated with an increased incidence of GDM. As our supplementation data was grouped to cover the whole of pregnancy, it is possible that the association partially relates to women with diagnosed GDM wanting to try to improve their diets, and supplementing with multiple micronutrient was part of this. However, the vast majority of the women who supplemented their diets with multiple micronutrients in this analysis started this either prior to or early in pregnancy, and the significant association was still evident when excluding women who only started supplementing after their OGTTs had taken place. It seems, therefore, that the association with GDM, rather than resulting from the GDM, actually relates to its development. Although this analysis was limited by not having access to highly detailed nutritional status records, as far as we can tell, the risk that we observed was not confounded by diet, as the food frequency intakes (specific and limited as they were [9]) did not substantially change the relationship between multiple micronutrient supplementation in pregnancy and GDM development. Whilst we are not aware of any studies that have previously assessed the risk of GDM development in those women supplementing their diets with multiple micronutrients (in high-income countries), some of the individual micronutrients (such as folic acid and iron) have been associated with the development of GDM in other studies [18,19,20,21], or even with protection against its development (vitamin D [22] and, in contrast to other studies [20,21], folic acid [23]). Although links between multiple micronutrient supplementation and GDM in pregnancy have not been investigated previously, one study found a lack of association with pregnancy-induced hypertension [24], which we were able to confirm in the present analysis in terms of both pre-eclampsia and gestational hypertension.

Previous studies have shown associations between multiple micronutrient supplementation in pregnancy and increased size at birth, particularly birth weight and head circumference [25,26,27,28]. Results from the present analysis are consistent with these. Along with increases in birth weight and head circumference in women supplementing their diets with multiple micronutrients, for the first time, this analysis has shown increases in adiposity through skinfolds thickness measurements, albeit with possible regionalized differences due to the lack of association with quadriceps skinfolds thicknesses. Previously, no changes in offspring adiposity have been observed with maternal multivitamin supplementation [29], suggesting that the increased adiposity observed in the present analysis may relate to one or more of the minerals commonly found in multiple micronutrient preparations. The mechanism of how multiple micronutrient supplementation may lead to increased adiposity is not fully understood. However, results from the present analysis in the subset of women who underwent OGTTs suggest that it may involve the same mechanisms that lead to increased offspring size at birth in GDM pregnancies in this cohort [30], as nearly all of the associations between multiple micronutrient supplementation and increased size at birth appeared attenuated by adjustment for GDM. This attenuation was not evident for the association with head circumference, suggesting that other mechanisms may also be involved, although the overall effect sizes of these alternative mechanisms are likely to have been smaller.

This analysis was not able to, nor was it designed to, distinguish which of the constituents of multiple micronutrient preparations caused the increased size at birth and GDM risk, or whether there was an interactive effect of two or more of the micronutrients that did this. It also grouped together different multiple micronutrient brands, all of which contained at least three micronutrients and most of which contained at least 15 of them. The various preparations contained different micronutrients, different salts of the same micronutrients, or these micronutrients at different amounts. However, most pregnancy multiple micronutrient preparations are somewhat similar, and by grouping them together in this analysis, we had enough statistical power in the present analysis to detect differences in various indices of offspring size at birth and risk of maternal GDM. This analysis did not have access to whether the micronutrient supplementation was prescribed or unprescribed. However, sensitivity analyses that excluded women reporting anemia in pregnancy (who would have been prescribed with iron or folic acid) showed similar trends to those observed in the full analysis, suggesting that this may not have biased the results.

Another potential limitation of the analysis could be possible biases introduced by its recruitment and analytical strategies. However, while the participants of the current analysis were not individually matched, the clinical characteristics of the group that supplemented with multiple micronutrients were very similar to those that did not supplement. The clinical characteristics of the whole group of participants in the present analysis were also very similar to those of women recruited to the CBGS who were excluded from it. In turn, the clinical characteristics of women recruited to the CBGS were representative of the whole population of women attending the Rosie Maternity Hospital, Cambridge, with the exception that they tended to be a little bit older and were more likely to be primiparous [8]. The relatively older age may not have affected our analysis, however, as sensitivity analyses that excluded women above 30 years of age showed increased effect sizes of associations with skinfold thicknesses, as well as a significantly increased risk of GDM development in the younger women. Whilst the associations with skinfold thicknesses did not reach statistical significance, this may have been due to a lack of statistical power in the small number of younger women tested. A further limitation of the analysis could relate to biases introduced by missing data, the extent of which varied by measurement, as well as the unavailability of data pertaining to familial history of diabetes or previous GDM, both risk factors for the development of GDM. In this analysis, missing data were dealt with by case or listwise deletions in order to prevent the introduction of inaccuracies resulting from data imputation. However, this may have introduced biases which are unfortunately common in this type of cohort study [31].

Other limitations of the current analysis include the facts that the micronutrient supplementation was self-reported, and that limited account was taken as to when in pregnancy and for how long supplementation took place. However, at least as far as supplementation specifically with folic acid in pregnancy, self-reporting has been validated as accurate [32,33]. In women with at least 12 years of education, one cohort also found that self-reporting of iron supplementation in pregnancy displayed high specificity [34]. In the present analysis, all 566 participants who responded to a question about their education in their pregnancy questionnaire reported that they had at least 12 years of education (data not shown), suggesting that for self-reporting of iron supplementation at least, high specificity could also have been achieved in the present analysis. Although only limited use of the timing and length of multiple micronutrient supplementation was made in this study, more than 80% of the respondents who supplemented with multiple micronutrients did so for more than half the pregnancy, which suggests that misclassification (due to only supplementing for a short period of time) is unlikely to have significantly affected the results. Finally, the cohort was recruited at the beginning of this century, and differences in dietary trends between then and now may limit the relevance of the results to the present age. Indeed, we previously observed some temporal trends in pregnancy dietary habits over the course of the recruitment period in the women studied in the present analysis [9]. However, the effect sizes of these trends were very small, and even if they continued at the same rate after the recruitment period, would not have severely limited the relevance of the current analysis to the present age.

## 5. Conclusions

In conclusion, in this analysis, we found evidence that maternal micronutrient supplementation in pregnancy is associated with increased size at birth and adiposity in the offspring in a population from a high-income country. It was also associated with increased risk of developing GDM in the mother, and the mechanisms that link GDM with increased size at birth may be partially responsible for the increased size at birth and adiposity resulting from maternal micronutrient supplementation in pregnancy.

## Figures and Tables

**Table 1:** A comparison of the characteristics of CBGS maternal participants who supplemented their diets with multiple micronutrients during pregnancy and those that did not.

Maternal Characteristic	No Multiple Micronutrients	Multiple Micronutrients	*p*‑Value
Age (years)	33.5	33.5	0.7
	(33.1–33.8)	(33.2–33.9)	
	(*n* = 408)	(*n* = 477)	
Height (m)	1.65	1.66	0.04
	(1.65–1.66)	(1.66–1.67)	
	(*n* = 410)	(*n* = 493)	
Pre-pregnancy weight (kg)	65.8	66.7	0.3
	(64.4–67.1)	(65.5–68.0)	
	(*n* = 476)	(*n* = 403)	
Pre-pregnancy BMI (kg/m^2^)	24	24.1	0.9
	(23.6–24.5)	(23.7–24.5)	
	(*n* = 391)	(*n* = 468)	
Weight gain in pregnancy (kg)	8.1	8.8	0.2
	(7.3–8.8)	(8.1–9.5)	
	(*n* = 289)	(*n* = 351)	
Index of multiple deprivation	8.8	8.7	0.6
	(8.5–9.1)	(8.5–9.0)	
	(*n* = 439)	(*n* = 526)	
Smoked during pregnancy (yes/no)	15/419	15/502	0.6
Anemia (n yes/no)	7/406	16/407	0.2
Length of Pregnancy (weeks)	39.8	40	0.1
	(39.7–40.0)	(39.8–40.1)	
	(*n* = 434)	(*n* = 517)	

Data are mean (95% confidence interval) or number of participants.

**Table 2:** Associations between maternal micronutrients supplementation status in pregnancy and indices of offspring size at birth.

Measure	*N*	Standardized β	*p*‑Value
Weight	855	0.063 (0.005–0.123)	0.03
Length *	833	0.032 (−0.018–0.081)	0.2
Head Circumference *	834	0.053 (0.002–0.100)	0.04
BMI *	831	0.050 (−0.006–0.101)	0.08
Ponderal Index *	831	0.038 (−0.015–0.088)	0.2
Flank skinfold thickness *	833	0.067 (0.003–0.127)	0.04
Quadriceps skinfold thickness *	834	0.042 (−0.010–0.095)	0.2
Subscapular skinfold thickness *	833	0.068 (0.005–0.127)	0.03
Triceps skinfold thickness *	833	0.095 (0.030–0.155)	3.0 × 10^−3^

Standardized βs are presented as means (95% confidence intervals). All models adjusted for gestational age at birth, parity, smoking during pregnancy, offspring sex, and maternal pre-pregnancy BMI. * Models additionally adjusted for age at assessment.

**Table 3:** Subgroup comparison assessing the effect of GDM on associations between multiple micronutrient supplementation in pregnancy and size at birth.

Measure	*N*	Unadjusted for GDM		Adjusted for GDM	
		Standardized β	*p*‑Value	Standardized β	*p*‑Value
Weight	600	0.048 (−0.021–0.112)	0.2	0.031 (−0.037–0.095)	0.4
Length *	583	0.009 (−0.050–0.067)	0.8	0 (−0.058–0.059)	1.0
Head Circumference *	584	0.035 (−0.022–0.097)	0.3	0.036 (−0.022–0.098)	0.3
BMI *	582	0.046 (−0.021–0.109)	0.2	0.034 (−0.032–0.097)	0.3
Ponderal Index *	582	0.042 (−0.022–0.104)	0.2	0.034 (−0.029–0.097)	0.3
Flank skinfolds thickness *	584	0.040 (−0.038–0.112)	0.3	0.022 (−0.055–0.093)	0.6
Quadriceps skinfolds thickness *	585	0.042 (−0.022–0.106)	0.3	0.034 (−0.030–0.099)	0.4
Subscapular skinfolds thickness *	584	0.061 (−0.015–0.128)	0.1	0.052 (−0.025–0.119)	0.2
Triceps skinfolds thickness *	584	0.058 (−0.021–0.123)	0.1	0.050 (−0.029–0.116)	0.2

Values in the table only include data from pregnancies where GDM status was available. Standardized βs are presented as means (95% confidence intervals). All models adjusted for gestational age at birth, parity, smoking during pregnancy, offspring sex, and maternal pre-pregnancy BMI. * Models additionally adjusted for age at assessment.

**Table:** (**a**)

Pregnancy Outcome	*N* (yes/no)	Risk Ratio	*p*-Value
GDM	63/556	1.85 (1.11–3.10)	0.02
Pre-eclampsia	14/879	0.63 (0.22–1.79)	0.4
Gestational Hypertension	24/420	0.99 (0.45–2.15)	1.0
Low Birth Weight	23/859	0.91 (0.41–2.04)	0.8
SGA	4/878	2.50 (0.26–23.95)	0.4
Premature Birth	15/869	0.56 (0.20–1.56)	0.3

**Table:** (**b**)

Measure	*N*	Standardized β	*p*-Value
Weight	796	0.065 (0.004–0.118)	0.04
Length *	776	0.041 (−0.011–0.092)	0.1
Head Circumference *	776	0.045 (−0.001–0.099)	0.08
BMI *	774	0.043 (−0.014–0.096)	0.1
Ponderal Index *	774	0.027 (−0.027–0.080)	0.3
Flank skinfolds thickness *	776	0.074 (0.008–0.137)	0.03
Quadriceps skinfolds thickness *	776	0.055 (−0.003–0.108)	0.07
Subscapular skinfolds thickness *	776	0.066 (0.002–0.128)	0.045
Triceps skinfolds thickness *	776	0.098 (0.032–0.161)	3.3 × 10^−3^

Data are mean (95% confidence interval) or number of participants. All models in (b) adjusted for gestational age at birth, parity, smoking during pregnancy, offspring sex, and maternal pre-pregnancy BMI. * Models additionally adjusted for age at assessment.

**Table:** (**a**)

Pregnancy Outcome	*N* (yes/no)	Risk Ratio	*p*-Value
GDM	17/99	3.03 (1.05–8.75)	0.04
Pre-eclampsia	2/159	1.12 (0.07–17.57)	0.9
Gestational Hypertension	6/79	1.58 (0.34–7.35)	0.6
Low Birth Weight	3/158	0.56 (0.05–6.04)	0.6
SGA	1/160	N/A	
Premature Birth	2/159	N/A	

**Table:** (**b**)

Measure	*N*	Standardized β	*p*-Value
Weight	144	0.071 (−0.074–0.211)	0.3
Length *	141	0.067 (−0.056–0.184)	0.3
Head Circumference *	140	0.098 (−0.023–0.211)	0.1
BMI *	141	0.006 (−0.125–0.136)	0.9
Ponderal Index *	141	−0.021 (−0.139–0.100)	0.7
Flank skinfolds thickness *	141	0.090 (−0.057–0.233)	0.2
Quadriceps skinfolds thickness *	141	0.097 (−0.035–0.218)	0.2
Subscapular skinfolds thickness *	141	0.173 (0.029–0.314)	0.02
Triceps skinfolds thickness *	141	0.131 (−0.019–0.251)	0.09

Data are mean (95% confidence interval) or number of participants. N/A = not available. All models in (b) adjusted for gestational age at birth, parity, smoking during pregnancy, offspring sex, and maternal pre-pregnancy BMI. * Models additionally adjusted for age at assessment.

## Supplementary Materials

The following are available online at https://www.mdpi.com/2072-6643/12/11/3466/s1, Figure S1: Histograms showing the distributions of (a) when multiple micronutrient supplementation began relative to the start of pregnancy, and (b) the length of time that multiple micronutrient supplementation was taken during (and before) pregnancy, Table S1: Published nutrient constituents of the most commonly used multiple micronutrient supplements in this population, Table S2: Comparison of characteristics of those that were included in this present analysis and those that were excluded from it, Table S3: Effect of food frequency intakes as confounders in the logistic regression models describing the relationship between multiple micronutrient intake during pregnancy and risk of gestational diabetes in the Cambridge Baby Growth Study.
