# Supplementary material for: Multiple Micronutrient Supplementation during Pregnancy and Increased Birth Weight and Skinfold Thicknesses in the Offspring: The Cambridge Baby Growth Study
Source: Nutrients. 2020 Nov 12;12(11):3466. doi: 10.3390/nu12113466 (PMC7697774; doi:10.3390/nu12113466)
Supplement: Supplementary file 1 [file nutrients-12-03466-s001.pdf]

Supplementary Figure & Tables for Petry et al. "Multiple Micronutrient Supplementation During Pregnancy and Increased Birth Weight and Skinfold Thicknesses in the Offspring: the Cambridge Baby Growth Study"

Figure S1 Histograms showing the distributions of (a) when multiple micronutrient supplementation began relative to the start of pregnancy, and (b) the length of time that multiple micronutrient supplementation was taken during (and before) pregnancy.

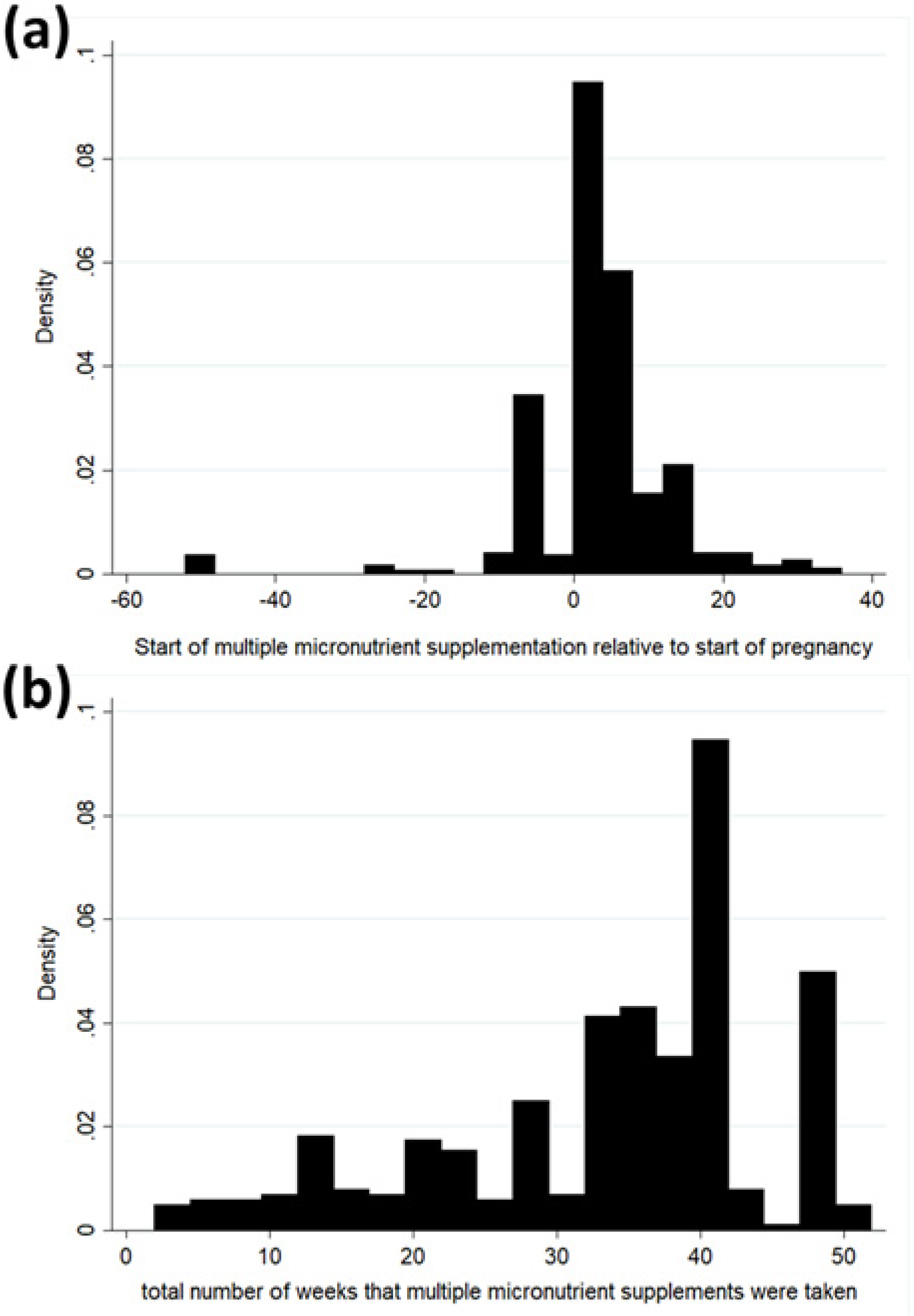

Table S1 Published nutrient constituents of the most commonly used multiple micronutrient supplements in this population.

**Multiple Micronutrient Preparation**	**Vitabiotics Pregnancare***	**Sanatogen Pronatal**	**Tesco Multiplus Pregnancy**
Vitamin D	10 µg (as D3 400 IU)	12.5 µg	10 µg
Vitamin E	4 mg (α-tocopherol equivalents)	10 mg (α-tocopherol)	12 mg
Vitamin K	70 µg	N/A	75 µg
Vitamin C	70 mg	100 mg	80 mg
Thiamin (Vitamin B1)	3 mg	1.6 mg	1.1 mg
Riboflavin (Vitamin B2)	2 mg	1.8 mg	1.4 mg
Niacin (Vitamin B3)	20 mg (niacin equivalents)	19 mg	16 mg
Vitamin B6	10 mg	2.6 mg	1.4 mg
Folic Acid	400 µg	400 µg	400 µg
Vitamin B12	6 µg	2.5 µg	2.5 µg
Biotin	150 µg	200 µg	50 µg
Pantothenic acid	6 mg	8.7 mg	6 mg
Calcium	N/A	170 mg	120 mg
Magnesium	150 mg	100 mg	60 mg
Iron	17 mg	14 mg	14 mg
Zinc	15 mg	7.5 mg	10 mg
Copper	1 mg	1 mg	1 mg
Selenium	30 µg	N/A	75 µg
Chromium	N/A	N/A	40 µg
Iodine	150 µg	N/A	150 µg
Natural mixed carotenoids	2 mg (β-carotene)	N/A	N/A
Phosphorus	N/A	133.3 mg	N/A
Manganese	N/A	1 mg	N/A

*refers to Pregnacare Original supplement preparations.
N/A = not available. IU = International Units.

Table S2 Comparison of characteristics of those that were included in this present analysis and those that were excluded from it.

Characteristic	Included in this analysis	Excluded from this analysis	*p*-value
Age (years)	33.5 (33.2—33.8) (n=885)	33.5 (33.1—33.9) (n=450)	0.9
Maternal pre-pregnancy BMI (kg/m^2)	24.1 (23.8—24.4) (n=859)	24.1 (23.6—24.6) (n=328)	0.9
Weight gain during pregnancy (kg)	8.466 (7.946—8.986) (n=640)	7.564 (6.705—8.424) (n=234)	0.08
Smoked during pregnancy (n yes/no)	30/921	56/648	<0.001
Gestational diabetes (n yes/no)	66/600	45/373	0.7
Gestational hypertension (n yes/no)	26/442	18/234	0.4
Preeclampsia (n yes/no)	14/956	8/1,248	0.06
Multifetal pregnancy (n twin pregnancies/singleton pregnancies)	0/970	21/1,214	<0.001
Gestational age at birth of offspring (weeks)*	39.9 (39.8—39.9) (n=951)	39.7 (39.6—39.8) (n=706)	0.08
Adjusted offspring birth weight (kg)**	3.476 (3.447—3.506) (n=855)	3.476 (3.428—3.525) (n=327)	1.0

Data are numbers of participants or mean (95% confidence interval)
*adjusted for twin pregnancies
**adjusted for gestational age at birth, parity, sex, maternal BMI and twin pregnancies

Table S3 Effect of food frequency intakes as confounders in the logistic regression models describing the relationship between multiple micronutrient intake during pregnancy and risk of gestational diabetes in the Cambridge Baby Growth Study.

**Food/Drink type**	**n**	**Relative Risk**	***p*-value**
None	666	1.86 (1.13—3.08)	0.02
Baked beans	660	1.89 (1.14—3.11)	0.01
Bean curd	657	1.88 (1.13—3.11)	0.01
Beer	636	2.00 (1.19—3.37)	9.2×10^{-3}
Canned fish	660	1.87 (1.13—3.08)	0.02
Canned meat	659	1.86 (1.13—3.07)	0.02
Canned (non-baked) beans	656	1.76 (1.06—2.92)	0.03
Chocolate	657	1.81 (1.10—3.00)	0.02
Cocoa	636	1.98 (1.17—3.34)	0.01
Coffee	652	1.92 (1.15—3.20)	0.01
Cola	636	2.01 (1.19—3.39)	9.1×10^{-3}
Dried fruit	658	1.92 (1.16—3.18)	0.01
Eggs	661	1.86 (1.12—3.07)	0.02
Fish	658	1.87 (1.13—3.08)	0.02
Fresh fruit	661	1.83 (1.11—3.03)	0.02
Fresh fruit juice	656	1.86 (1.12—3.07)	0.02
Fresh green vegetables	658	1.83 (1.11—3.03)	0.02
Fresh non-green vegetables	659	1.86 (1.12—3.07)	0.02
Frozen vegetables	659	1.86 (1.13—3.08)	0.02
Hard cheese	661	1.86 (1.12—3.07)	0.02
Liver	660	1.84 (1.11—3.04)	0.02
Meat	660	1.86 (1.12—3.07)	0.02
Organic food	657	1.86 (1.12—3.08)	0.02
Poultry	659	1.85 (1.12—3.06)	0.02
Pulses	659	1.78 (1.07—2.97)	0.03
Salad	651	1.82 (1.10—3.01)	0.02
Shellfish	653	1.81 (1.09—2.99)	0.02
Soft cheese	645	1.84 (1.11—3.05)	0.02
Soya	658	1.81 (1.09—2.99)	0.02
Spirits	636	1.83 (1.09—3.06)	0.02
Tea	649	1.96 (1.16—3.31)	0.01
Tinned fruit	658	1.85 (1.12—3.06)	0.02
Tinned vegetables	661	1.89 (1.15—3.13)	0.01
White fish	657	1.83 (1.10—3.02)	0.02
Wine	643	1.93 (1.14—3.25)	0.01
Yogurt	658	1.81 (1.09—2.99)	0.02

Data are either individual numbers or means (95% confidence intervals).
